# Infectious causes of pregnancy loss in cattle: a review of loss intensity and major reproductive pathogens involved

**DOI:** 10.1590/1984-3143-AR2025-0064

**Published:** 2026-04-20

**Authors:** Rodrigo de Morais, Giovanna Meireles Borges, Moyses dos Santos Miranda, Luiz Francisco Machado Pfeifer, Rinaldo Batista Viana, Bruno Moura Monteiro

**Affiliations:** 1 Programa de Pós-graduação em Reprodução Animal na Amazônia – ReproAmazon, Instituto de Saúde e Produção Animal – ISPA, Universidade Federal Rural da Amazônia – UFRA, Belém, PA, Brasil; 2 Instituto Federal de Educação, Ciência e Tecnologia do Pará – IFPA, Marabá, PA, Brasil; 3 Graduação em Medicina Veterinária, Instituto de Saúde e Produção Animal – ISPA, Universidade Federal Rural da Amazônia – UFRA, Belém, PA, Brasil; 4 Programa de Pós-graduação em Reprodução Animal na Amazônia – ReproAmazon, Instituto de Medicina Veterinária – IMV, Universidade Federal do Pará – UFPA, Castanhal, PA, Brasil; 5 Empresa Brasileira de Pesquisa Agropecuária – EMBRAPA, Porto Velho, RO, Brasil

**Keywords:** abortion, embryonic mortality, reproductive disease, reproductive failure, distribution of losses

## Abstract

Pregnancy losses represent one of the main economic problems in cattle farming, where up to half of these losses can be associated with infectious diseases. Early identification of risk factors is essential to prevent reproductive and improve productivity in both beef and dairy systems. However, there are still a few studies in literature that relate etiological agents and the moment of gestation in which these reproductive failures occur. Embryonic and fetal losses vary widely across studies due to differences in diagnostic methods, herd management, pathogen exposure, and environmental conditions. This review summarizes the current scientific evidence on the intensity of pregnancy loss in cattle caused by infectious agents, relating to the most affected gestational stages and the most relevant pathogens. The main etiological agents identified were *Neospora caninum*, bovine viral diarrhea virus (BVDV), bovine herpesvirus type 1 (BoHV-1), *Leptospira* spp. and *Campylobacter fetus*. These infections compromise the reproductive efficiency of herds, causing infertility, embryonic mortality and abortions. *Neospora caninum* was the main agent associated with pregnancy losses, with abortions reported between the third and ninth months of gestation. BVDV was the second most frequently associated agent. Most pregnancy losses occurred between the second and third thirds of gestation, which makes early diagnosis of reproductive failures and the adoption of effective preventive measures difficult. The intensity and frequency of losses varied according to the agent involved, the geographic region and the type of production system. The findings of this review reinforce the need for continuous reproductive monitoring, especially with the use of pregnancy diagnostics at the end of the breeding season, in addition to the implementation of efficient biosecurity programs on properties.

## Introduction

Pregnancy loss is one of the reproductive failures with the greatest economic impact in livestock production (Reese et al., 2020; Prado et al., 2024). In extensive production systems, these losses tend to be underestimated, prolong the interval between conceptions, and reduce the profitability of the activity (Santos et al., 2004; Reese et al., 2020; Consentini et al., 2023). In dairy herds, the loss associated with each pregnancy loss has been estimated at US$ 555, varying according to the stage of gestation and the timing of lactation (De Vries, 2006). In beef cattle, a 1% reduction in pregnancy rate per cow exposed to artificial insemination (AI) results in an average loss of US$ 6.25 (Mercadante et al., 2020), as recently reviewed by Sartori et al. (2025).

In addition to the economic impact, pregnancy loss represents a relevant health challenge, especially because the diagnostic capacity to identify infectious causes varies widely among regions, laboratories, and the methods used (Herlina et al., 2025). This limitation contributes to the wide variation observed in the reported rates of pregnancy loss. (Diskin et al., 2016). The causes affecting pregnancy maintenance are multifactorial and include physiological and nutritional aspects, placental competence, management practices, and health status (Baruselli et al., 2017; Alves et al., 2021; Hecker et al., 2023). It is estimated that up to 50% of embryonic losses are related to infectious diseases (Aono et al., 2013). Among the infectious agents, bovine herpesvirus type 1 (BoHV-1), bovine viral diarrhea virus (BVDV), the bacteria *B. abortus* and *Leptospira* spp., and the protozoan *Neospora caninum* are considered the main pathogens (Aono et al., 2013; Hecker et al., 2023).

Although pregnancy losses caused by infectious agents have significant economic relevance (Reese et al., 2020), studies that associate their occurrence with the stage of gestation in cattle remain scarce. Furthermore, most existing studies focus on isolated infectious agents or specific gestational phases, without providing a comparative view of losses throughout the entire gestation period (Morrell et al., 2019; Van Loo et al., 2021; Şevik, 2021; Thomas et al., 2022).

Given this scenario, it is essential to deepen the understanding of the distribution of reproductive losses throughout gestation and to relate them to the potentially involved etiological agents (Hecker et al., 2023; Aymée et al., 2024; Prado et al., 2024). Therefore, our objective was to conduct a comprehensive literature review on reproductive losses caused by infectious agents in cattle. Additionally, this study aimed to associate infectious agents with the timing of pregnancy losses.

## Methodology of literature review

This narrative review focused exclusively on the infectious causes of gestational loss in cattle, including early and late embryonic loss and fetal loss. A comprehensive literature search was conducted in the PubMed, Scopus, SciELO, and Web of Science databases, using Boolean operators (AND, OR) and combinations of keywords related to infectious reproductive failure (“infectious agents AND gestational loss”, “cattle AND gestational loss”, “embryonic loss OR fetal loss”, “cattle AND reproductive infection”).

Only peer-reviewed studies addressing infectious gestational loss in cattle were included, spanning publications from 1981 to 2025, totaling 104 articles. In addition to etiological reports, articles describing the physiological mechanisms of pregnancy establishment, embryonic and fetal development, and the pathophysiology underlying infectious pregnancy loss were also considered, whereas non-infectious causes were excluded. For the section that synthesizes specific pathogen patterns and the intensity of pregnancy loss throughout gestation, the data were refined to studies published between 2014 and 2024, resulting in 29 eligible articles, encompassing 12.235 reported cases, which provided a standardized dataset to estimate the intensity of loss associated with the main infectious agents.

## Pregnancy loss in cattle

Pregnancy loss can occur at different stages of pregnancy and from various causes, but it clearly has a negative impact on reproductive performance in livestock, especially in ruminants (Wiltbank et al., 2016). The most critical period of reproductive failure occurs during the first month of pregnancy. High-producing dairy cattle experience the highest rates of these losses, approximately 60% of conceptions fail to establish pregnancy and survive beyond the 30^th^ day of gestation (Wiltbank et al., 2016; Mathew et al., 2022). A similar scenario occurs in beef cattle, where more than 40% of conceptions can be lost due to embryonic mortality (Reese et al., 2020).

Regardless of the productive aptitude of cattle, most reproductive losses happen in the first 8 days of the embryo's life (Santos et al., 2004; Diskin et al., 2011; Wiltbank et al., 2016; Smith et al., 2022). Moreover, embryos produced by assisted reproductive technologies, such as *in vitro* fertilization and somatic cell nuclear transfer, are more susceptible to embryonic and fetal mortality compared to their counterparts developed *in vivo* (Mathew et al., 2022; Sartori et al., 2025).

According to Wiltbank et al. (2016) gestational losses can be divided into 4 periods, when considering the intensity of the losses. It is important notice that losses can also be divided according to the development of the conceptus (embryo or fetus), but this will be discussed below. The first period occurs during the first week after service/mating, with a lack of fertilization or death of the newly formed embryo, producing large pregnancy losses, particularly under specific environmental and hormonal conditions. In general, 20% to 50% of pregnancy failures during this crucial period result in a restart of the estrus cycle, as if gestation had not begun.

The second crucial period, from days 8 to 28, covers embryo elongation and the classic maternal-fetal recognition period, with average losses of approximately 30%, but with surprising variation between farms (25%-41%) (Smith et al., 2022). The third crucial period occurs during the second month of gestation, from days 28 to 60, with losses of between 8 and 14% (Couto et al., 2019). Finally, a fourth period of pregnancy losses occurs during the third month of gestation, with reduced losses (~2%) compared to the first three periods, but can be high in some cows, particularly those with reproductive diseases (Couto et al., 2019; Smith et al., 2022).

Some research demonstrates how that the ovulation rate in *Bos indicus* beef cows is up to 90.9% in follicles with a diameter of 13mm (Pugliesi et al., 2016) and show high fertilization which generally exceeds 90% (Diskin et al., 2011, 2016). These findings indicate that the pregnancy rate at the end of the breeding season is directly related to gestational losses.

The inflammatory condition of the uterus at the start of the timed artificial insemination (TAI) protocol has been reported as one of the factors for a lower pregnancy rate in beef cattle (Andrade et al., 2021) when the polymorphonuclear count was > 4.75%. However, conditions of subclinical endometritis did not affect pregnancy loss between 30 and 100 days of gestation (Oliveira et al., 2022). Considering that pregnancy loss occurs mainly in the first month of gestation (Wiltbank et al., 2016; Franco et al. 2020; Smith et al., 2022), the experimental design may not have been able to measure the influence on early embryonic mortality. From this perspective, further studies are needed to analyse the relationship between subclinical endometritis and gestational loss up to 28 days.

The modernization of synchronization protocols to improve female fertility is frequently proposed. Consentini et al. (2023) evaluated different factors related to pregnancy loss in *Bos indicus* beef cattle and observed that treatment with GnRH at the time of artificial insemination or PGF2a at D0 did not influence pregnancy loss in beef cows submitted to protocols based on E2 and P4. In addition, there was no effect of the presence of corpus luteum at D0, body condition score, bull or number of services (first TAI or resynchronization).

Nevertheless, conflicting results have been found in relation to pregnancy loss and estrus status at TAI. Consentini et al. (2023) observed that multiparous and primiparous cows that did not express estrus at the end of the TAI protocols had greater pregnancy loss (13.5% vs. 9.7%). On the other hand, early embryonic mortality (21 to 31 days, estrus, 4.3% vs. no estrus, 6.8%) and late embryonic/early fetal mortality (31 to 60 days, estrus, 8.27% vs. 6.17% no estrus) was not affected by the manifestation of estrus at the end of TAI in beef cows (Franco et al., 2020). These findings suggest that, although estrus expression may be associated with greater reproductive competence in some contexts, its direct impact on pregnancy loss remains inconsistent across studies, reflecting the multifactorial influence on fertility after TAI.

In a recent study, Prado et al. (2024) characterized the reproductive losses at different gestational periods of *Bos indicus* females submitted to TAI. Precocious heifers inseminated at 12 months exhibited the highest total pregnancy loss from day 30 to calving (28.4%; 177/642), followed closely by conventional heifers inseminated at 24 months (27.1%; 167/645). In contrast, primiparous (16.4%; 71/391) and multiparous cows (13.0%; 64/486) had significantly lower losses (P < 0.05), with similar performance between these adult groups. Curiously, many losses were due to fetal mortality between day 60^th^ and 150^th^ of gestation (10.2%), compared to day 30-60 of gestation (6.0%) and from day 150 of gestation to calving (7.4%). This concentration of mid-gestation losses may indicate a relevant contribution of infectious etiologies. Despite their importance, studies that comprehensively evaluate pregnancy loss dynamics throughout the entire gestational period remain limited in the literature.

## Early and late embryonic loss

Embryonic mortality, if classified strictly according to physiological events during gestation, should refer to losses during the embryonic period, which runs from conception to the end of the differentiation phase, around the 42^nd^ day of gestation in cattle (Santos et al., 2004; Mathew et al., 2022).

Early embryonic mortality, defined as the death or loss of embryos before the 28^th^ day of gestation, is an important factor in reproductive failure (Franco et al., 2020), while late mortality covers the period from 28 to 42 days (Wiltbank et al., 2016).

Studies compiled in the meta-analysis by Reese et al. (2020) indicate that most pregnancy losses in beef cattle occur during early embryonic development (first month of gestation, 47.9%), whereas losses during late embryonic and early fetal stages (up to ~100 days, 5.8%) are comparatively lower. A more recent meta-analysis by Albaaj et al. (2022) reported mean loss rates of 13% for late embryonic mortality, 7% for early fetal mortality, and only 2% for fetal losses between 60 and 90 days of gestation, reinforcing the declining pattern of losses as gestation progresses. Field studies support this trend: in grazing Nelore cows submitted to TAI, Couto et al. (2019) reported 8% loss between 30 and 60 days and 2% between 60 and 90 days. Similarly, Smith et al. (2022) observed that 40–50% of total pregnancy losses occurred within the first month of gestation, further highlighting the disproportionate contribution of early embryonic mortality.

Although most evidence points to early embryonic loss as the predominant component of overall reproductive failure, data from confined dairy herds show that substantial mid- and late-gestation losses may also occur. In a recent study, lactating Holstein cows raised under tropical conditions and previously vaccinated against BoHV-1, BVDV and *Leptospira* spp. exhibited a pregnancy loss rate of 28.5% after day 31 of gestation (162/568). Most losses occurred after day 62 (17.8%) (Munhoz et al., 2025). Multiparous cows showed greater losses than primiparous cows between days 31 and 62 (17.1% vs. 9.5%) and from day 120 to calving (15.4% vs. 7.7%). Although the authors attributed these losses primarily to heat stress, the study did not screen for infectious agents capable of inducing mid-to-late gestational mortality. This limitation is noteworthy because multiparous cows typically have greater cumulative exposure to abortifacient pathogens such as *N. caninum*, which predominantly induces fetal loss after mid-gestation (Van Loo et al., 2021). Supporting this concern, Lefkaditis et al. (2020) reported that 28.3% of abortions (52/184) in confined Holstein cows occurred in *N. caninum* positive animals, reinforcing the pathogen’s relevance in intensive dairy systems.

Complementary evidence from beef cattle further illustrates the progressive decline in loss rates as gestation advances. In beef cows submitted to early resynchronization, two days after TAI, pregnancy loss was reported at 13.4% between D20 and D30, 2.8% between D30 and D60 and 1.9% between D60 and D90 (Pugliesi et al., 2019). Similar results were observed by Prado et al. (2024) in multiparous cows, with losses of 3.7% between 30 and 60 days.

## Fetal loss

The fetal phase follows the embryonic phase, during which diseases and/or toxins are often the cause of pregnancy failure in this period (Santos et al., 2004). It has been reported that fetal mortality occurs in less than 10% of pregnancies during this period (Smith et al., 2022).

Nevertheless, differences between categories are evident. Heifers tend to experience greater gestational losses than cows, with rates that can be twice as high as those observed in multiparous cows. Prado et al. (2024) reported losses of 21.1% in heifers and 9.44% in cows between day 60 of gestation and calving.

Research indicates that the lower serum concentrations of pregnancy-associated glycoproteins observed in heifers, compared to calving cows, may contribute to the higher rates of pregnancy loss (Prado et al., 2024). These glycoproteins are used as a marker of placental function in cattle (Wallace et al., 2015), suggesting inadequate placental development due to insufficient sexual maturation and uterine receptivity in nulliparous heifers (Davenport et al., 2023).

## Infectious pregnancy losses in cattle

The major infectious causes of pregnancy loss in cattle are related to bacterial, viral, and protozoan agents, which can affect pregnancy at different stages, either through prior infections or those contracted during gestation. These agents represent a significant concern for livestock production, being responsible for silent and often underreported gestational losses (Van Loo et al., 2021; Mee, 2023).

We would like to highlight the complexity of determining the causative agents of reproductive failure. For instance, a study evaluating 4,006 cases of infectious abortions was able to diagnose only 39% of the cases (Van Loo et al., 2021). In Brazil, a study analyzing 490 bovine fetuses at the pathology department of the Federal University of Rio Grande do Sul (UFRGS) identified specific causes of abortion in only 46.7% of cases (Antoniassi et al., 2013). Comparable findings were reported by Morrell et al. (2019) in Argentina, with a diagnostic rate of 52%, and more recently, 53% in Uruguay (Macías-Rioseco et al., 2020). These consistently low diagnostic rates highlight the limitations of traditional approaches and reinforce the need for more sensitive and integrative tools.

Recent diagnostic advances have expanded the capacity to identify infectious pregnancy losses in cattle, overcoming limitations of classical approaches that still leave more than 50% of cases without a defined etiology (Herlina et al., 2025). The adoption of panels combining ELISA/IFAT (Indirect Fluorescent Antibody Test), qPCR (uantitative Polymerase Chain Reaction), and multiplex PCR has increased the simultaneous detection of *N. caninum*, *Brucella abortus*, *Leptospira* spp., BoHV-1, BVDV, and *Coxiella burnetii* in maternal–fetal samples (Herlina et al., 2025). Metagenomics (16S/18S rRNA and shotgun), in turn, offers the most comprehensive approach, with high sensitivity and specificity for identifying emerging pathogens, non-cultivable microorganisms, and complex coinfections, although it is still limited by cost and analytical demands (Herlina et al., 2025).

Complementarily, genome-wide association studies (GWAS – Genome-Wide Association Study) have revealed loci and biological pathways associated with abortion, indicating that genetic markers may in the future be integrated into diagnostic protocols and preventive management strategies (Sigdel et al., 2021; Suarez et al., 2024; compiled in Herlina et al., 2025).

We reviewed the literature published over the past 10 years (2014–2024) on gestational losses in cattle caused by infectious agents, focusing on data regarding prevalence, gestational age, and herd health status (Table 1). The results indicate that *N. caninum* is the agent most frequently associated with high abortion rates, ranging from 5.4% to 49.4%, and has been identified across multiple countries in the Americas, Europe, Africa, and Asia, primarily affecting dairy herds (Delooz et al., 2017; Açici et al., 2019). Losses attributed to this protozoan occur predominantly between the third and ninth months of gestation, with notably high abortion rates reported in Brazil (44.4%), Turkey (49.4%), and Iran (30.47%) (Pessoa et al., 2016; Açici et al., 2019; Kaveh et al., 2017). In line with these findings, recent reviews indicate that approximately 42% of bovine abortions are of infectious origin, with *N. caninum* consistently emerging as the main etiological agent worldwide (Mee, 2023; Hecker et al., 2023).

**Table 1:** Main infectious causes of abortions and percentage of gestational losses in cattle diagnosed in the last 10 years (2014-2024).

Abortion causes	Country	Pregnancy loss (%)	Month of gestation	Aptitude	Breed	N
*Brucella abortus*						
Derdour et al. (2017)	Algeria	3.06	ND	Dairy	ND	360
Morrell et al. (2019)	Argentina	6.65	ND	DY/BF	ND	150
Zhang et al. (2020)	China	6.76	ND	Dairy	ND	1406
Jonker and Michel (2021)	S. Africa	7.3	ND	DY/BF	ND	193
Ntivuguruzwa et al. (2022)	Rwanda	10.5	ND	Dairy	ND	19
Thomas et al. (2022)	Tanzania	1.4	ND	ND	ND	71
*Leptospira* spp.						
Clothier and Anderson (2016)	USA	0.7	6° - 9°	DY/BF	ND	709
Derdour et al. (2017)	Algeria	3.89	ND	Dairy	ND	360
Delooz et al. (2017)	Belgium	0.5	ND	DY/BF	ND	368
Kaveh et al. (2017)	Iran	14.06	ND	Dairy	ND	128
Vidal et al. (2017)	Switzerland	21.4	ND	DY/BF	ND	249
Morrell et al. (2019)	Argentina	7.33	ND	DY/BF	ND	150
Kim et al. (2024)	South Korea	3.6	ND	DY/BF	Hanwoo/ Holstein	360
*Campylobacter fetus sp*						
Clothier and Anderson (2016)	USA	0.7	6º - 9º	DY/BF	ND	709
Morrell et al. (2019)	Argentina	9.33	ND	DY/BF	ND	150
Macías-Rioseco et al. (2020)	Uruguay	2	ND	Dairy	ND	102
BVDV						
Clothier and Anderson (2016)	USA	1.7	6º - 9º	DY/BF	ND	709
Cvetojević et al. (2016)	Serbia	2	5º and 8°	Dairy	Holstein-Frisian	100
Wilson et al. (2016)	Canada	3.36	ND	DY/BF	ND	236
Derdour et al. (2017)	Algeria	1.39	ND	Dairy	ND	360
Delooz et al. (2017)	Belgium	3.5	ND	DY/BF	ND	368
Kaveh et al. (2017)	Iran	20.31	ND	Dairy	ND	128
Díaz-Cao et al. (2018)	Spain	11.1	ND	Dairy	ND	16
Morrell et al. (2019)	Argentina	1.33	ND	DY/BF	ND	150
Macías-Rioseco et al. (2020)	Uruguay	2.9	ND	Dairy	ND	102
Szeredi et al. (2020)	Hungary	9	ND	DY/BF	ND	387
Van Loo et al. (2021)	Belgium	3.5 - 5.4	3° - 9º	DY/BF	Holstein/Belgian Blue	4006
Şevik (2021)	Turkey	11	ND	DY/BF	ND	553
Kim et al. (2024)	South Korea	18.1	ND	DY/BF	Hanwoo/ Holstein	360
BoHV-1						
Clothier and Anderson (2016)	USA	3.5	3° - 9°	DY/BF	ND	709
Wilson et al. (2016)	Canada	0.84	ND	DY/BF	ND	236
Derdour et al. (2017)	Algeria	0.55	ND	Dairy	ND	360
Kaveh et al. (2017)	Iran	13.28	ND	Dairy	ND	128
Morrell et al. (2019)	Argentina	2.0	ND	DY/BF	ND	150
Thomas et al. (2022)	Tanzania	4.1	ND	ND	ND	71
*Neospora caninum*						
Clothier and Anderson (2016)	USA	9.3	3º - 9º	DY/BF	ND	709
Cvetojević et al. (2016)	Serbia	1.0	7º	Dairy	Holstein-Frisian	100
Pessoa et al. (2016)	Brazil	44.4	ND	Dairy	ND	1273
Wilson et al. (2016)	Canada	18.2	3º - 6º	DY/BF	ND	236
Delooz et al. (2017)	Belgium	5.4	ND	DY/BF	ND	368
Derdour et al. (2017)	Algeria	15	ND	Dairy	ND	360
Kaveh et al. (2017)	Iran	30.47	ND	Dairy	ND	128
Díaz-Cao et al. (2018)	Spain	11.1	ND	Dairy	ND	16
Moroni et al. (2018)	Chile	14.9	ND	Dairy	ND	296
Morrell et al. (2019)	Argentina	14.67	ND	DY/BF	ND	150
Açici et al. (2019)	Turkey	49.4	3º - 9º	DY/BF	ND	89
Serrano-Martínez et al. (2019)	Peru	15	2º - 9º	Dairy	Holstein	219
Macías-Rioseco et al. (2020)	Uruguay	29	3º - 6º	Dairy	ND	102
Lefkaditis et al. (2020)	Greece	8	ND	Dairy	Holstein-Frisian	875
Szeredi et al. (2020)	Hungary	13	ND	DY/BF	ND	387
Wolf-Jäckel et al. (2020)	Denmark	19	ND	Dairy	ND	162
Van Loo et al. (2021)	Belgium	13.3 - 21.7	3º - 8º	DY/BF	Holstein/Belgian Blue	4006
Perotta et al. (2021)	Brazil	15.43	ND	Dairy	Holstein	75
Villa et al. (2021)	Italy	27.8	ND	Dairy	Holstein- Friesian	198
Thomas et al. (2022)	Tanzania	12.7	ND	ND	ND	71
Selim et al. (2023)	Egypt	35.5	ND	Dairy	ND	310
Kim et al. (2024)	South Korea	2.2	ND	DY/BF	Hanwoo/ Holstein	360

N: sample size. Aptitude: DY: dairy; BF: beef. ND: not described; USA: United States of America; S. Africa: South Africa. Percentages represent pregnancy loss reported in each original study. The table compiles peer-reviewed publications included in this review and reflects variability across production systems and geographic regions.

The BVDV emerges as the second most relevant infectious pathogen linked to gestational loss, with reported prevalence values ranging from 1.39% to 20.3% in both dairy and beef systems (Wilson et al., 2016; Kaveh et al., 2017). Consistent with this epidemiological pattern, BVDV is also recognized as one of the most frequent viral causes of abortion worldwide, and like *N. canimun*, is commonly associated with outbreaks rather than sporadic cases (Reese et al., 2020; Mee, 2023). Its impact is particularly pronounced in herds with inadequate biosecurity or persistent infection dynamics (Herlina et al., 2025).

Bovine herpesvirus type 1 (BoHV-1), although widely distributed, generally presents lower abortion rates, typically below 4.5%, according to studies from North America, Africa, and South America (Clothier and Anderson, 2016; Wilson et al., 2016; Derdour et al., 2017; Morrell et al., 2019; Thomas et al., 2022). Other viral pathogens such as *Coxiella burnetii* and *Chlamydia* spp. have been increasingly reported, although their contribution varies geographically and is strongly dependent on diagnostic capacity (Reese et al., 2020).

*Brucella abortus*, *Leptospira* spp., and *Campylobacter fetus* have even lower point prevalences (typically below 10%), with exceptions such as leptospirosis cases in Switzerland (21.4%; Vidal et al., 2017) and Iran (14.06%; Kaveh et al., 2017). Among bacterial agents, brucellosis remains a significant cause of abortion in countries where eradication programs have not been fully implemented (Otter, 2020; Mee, 2023; Poester et al., 2013).

Although less frequent than protozoal, viral, or bacterial causes, fungal abortions constitute a relevant component of infectious pregnancy losses in cattle (Andrade and Simões, 2024). Mycotic abortion has been associated with more than 35 fungal species (Van Kuijk et al., 2015), with *Aspergillus fumigatus* being the most frequently identified agent (Andrade and Simões, 2024). It is estimated that approximately 60% of cases are related to *A. fumigatus*, while about 20% involve zygomycetes such as *Absidia*, *Mucor*, *Rhizomucor*, *Geotrichum*, and *Rhizopus* (Eidi et al., 2024). Epidemiological reports confirm their relevance: in southern Brazil, *Aspergillus* accounted for 16.7% (4/24) of the investigated abortions (Henker et al., 2022), whereas in Iran fungal contamination was detected in 21% (42/200) of aborted fetuses (Eidi et al., 2024).

These saprophytic organisms are widely distributed in the environment and are frequently associated with poor-quality silage, spoiled feed, or contaminated soil (Walker, 2007). Fetal infection generally occurs through hematogenous dissemination after entry of the agent via the respiratory or gastrointestinal routes, resulting in necrotizing placentitis and fetal death (Austin, 2021). Most mycotic abortions are reported between the sixth and eighth months of gestation, although sporadic cases may occur at other stages (Austin, 2021; Henker et al., 2022).

It is important to note that fetal and perinatal mortality can result from the involvement of multiple infectious and/or non-infectious agents within a single case (co-mortality or polymicrobial infection) (Macías-Rioseco et al., 2020; Mee, 2020). In a recent study, Van Loo et al. (2021) reported coinfections in 8% of diagnosed cases. Infections such as BVDV, which are known to cause immunosuppression (Roth et al., 1981), may predispose cows and fetuses/neonates to opportunistic bacterial or saprophytic fungal infections, or even to the reactivation of latent *N. caninum* bradyzoites (Marugan-Hernandez, 2017). The high occurrence of mixed infections observed by Sarangi et al. (2021) in 43.75% of abortion cases in southern India reinforces this pattern. Recognition of this multifactorial scenario has important diagnostic implications, as investigations focused on a single agent tend to underestimate the complexity of the infectious process and contribute to the high proportion of abortions without a defined etiology (Herlina et al., 2025).

Most studies did not specify the gestational age at which losses occurred, making it difficult to assess the impact of infectious agents throughout pregnancy. We recommend that future studies report the gestational age of reproductive losses, as this information could support the development of more targeted prevention strategies. The available data indicate a higher frequency of abortions during the middle and final thirds of gestation, particularly in cases involving viral agents and *N. caninum* (Cvetojević et al., 2016; Clothier and Anderson, 2016; Açici et al., 2019; Van Loo et al., 2021).

According to surveys conducted in both beef and dairy cattle, the rate of gestational loss can vary between 5% and 20%, depending on the stage of pregnancy and the infectious agent involved (Mee et al., 2023). A study encompassing 56,000 diagnoses found that infections may account for up to 30% of gestational losses during the second and third trimesters of pregnancy (Mee, 2023). In herds affected by *Campylobacter fetus*, the abortion rate can reach up to 10% (Reese et al., 2020). Leptospirosis is also frequently associated with reproductive losses, particularly in tropical regions, and may cause gestational losses ranging from 0.5% to 21% in severe outbreaks (Delooz et al., 2017; Vidal et al., 2017; Morrell et al., 2019).

*Neospora caninum* is the most important protozoan associated with reproductive infectious diseases in cattle. Since its identification as a cause of abortion in 1989, it has become the most frequently reported infectious agent responsible for bovine abortion worldwide (Thilsted and Dubey, 1989). It is more commonly diagnosed in dairy cows than in beef cattle, likely due to their closer contact with domestic canids (Wilson et al., 2016; Otter, 2020; Van Loo et al., 2021).

Fetuses aborted due to *N. caninum* infection are typically between 3 and 9 months of gestational age and often exhibit characteristic lesions in fetal tissues (Van Loo et al., 2021). These include necrotizing encephalitis, myocarditis, hepatitis, and necrotizing lesions in the lungs and kidneys, as well as placentitis, which facilitates clinical diagnosis (Dubey et al., 2006; Pescador et al., 2007).

Canids play a crucial role in the life cycle of this parasite, as they shed infective oocysts in their feces, thereby contaminating the farm environment (Donahoe et al., 2015). Currently, there are no commercially available vaccines against *N. caninum* on the international market. The only vaccine previously developed (Bovilis Neoguard®) was withdrawn due to its low efficacy (Weston et al., 2012). However, one study reported that a fresh, live *N. caninum* vaccine significantly reduced the abortion rate in dairy cows (Mazuz et al., 2015).

The main viral causes of abortion in cattle are BVDV and BoHV-1. Although some European countries have successfully eradicated these infections, they continue to pose significant challenges globally. Current data suggest that BVDV is associated with 2% to 6% of abortions (Mee, 2023), although rates as high as 20.31% have also been reported (Kaveh et al., 2017). The most abortion-prone period of gestation appears to be between the sixth and seventh months, with a higher incidence observed in dairy cows (5.2%) compared to beef cows (3.9%) (Van Loo et al., 2021).

Reproductive losses due to BVDV infection depend on the stage of gestation at which the infection occurs (Mee et al., 2023). In general, earlier the infection takes place during gestation, more severe the consequences are for the fetus, including a higher risk of embryonic mortality and abortion during the first month of pregnancy (Schweizer and Peterhans, 2014). These outcomes may lead to temporary infertility and return to estrus. Infections occurring before the development of fetal immune competence (between days 40 and 150 of gestation) can result in immune tolerance to the virus and the birth of persistently infected (PI) animals (Knapek et al., 2020).

On the other hand, fetal infections that occur after the maturation of the immune system led to the upregulation of genes associated with the adaptive immune response, without significant alterations in innate immune mechanisms (Knapek et al., 2020). This immune activation may result in the birth of either clinically normal or weak calves (Grooms, 2006), or in some cases, trigger fetal inflammatory response syndrome, which can lead to multiple organ failure and fetal death (Jawor et al., 2021).

BoHV-1, in turn, is associated with a higher incidence of abortion after the fourth month of gestation (Maresca et al., 2018). Clinical signs may or may not be present in affected herds and can include infectious bovine rhinotracheitis, vulvovaginitis, and conjunctivitis. The infection may also result in fetal mummification, stillbirth, or the birth of weak calves (Ata et al., 2012; Maresca et al., 2018).

Abortions caused by infectious bovine rhinotracheitis (IBR; BoHV-1) were evaluated by Khaneabad et al. (2023), who reported a fetal loss rate of 13.8% in herds with a seroprevalence of 96.7%. Similarly, Wedajo et al. (2021) observed a gestational loss rate of 13.9% in seropositive dairy cows. Moreover, their study found that the conception rate at first service was significantly higher in seronegative cows (74.4%) compared to seropositive cows (25.6%), indicating a direct impact of BoHV-1 infection on fertility.

It is important to note that gestational losses caused by IBR also affect fertility and oocyte quality in infected cows (Miller et al., 1988). Ovarian infection by the virus can lead to corpus luteum necrosis and degeneration of developing follicles, resulting in low-viability oocytes (Ata et al., 2012; Graham, 2013). These effects contribute not only to the occurrence of abortion but also to a prolonged impairment of the female's reproductive capacity.

The bacterial agents most associated with pregnancy loss are *Brucella abortus* and *Leptospira* spp. These two zoonotic pathogens share similar characteristics, particularly their capacity to induce abortion, especially during the second and third trimesters of gestation (Grooms, 2006).

*Brucella abortus* is the primary bacterium responsible for cases of bovine brucellosis, leading to spontaneous abortions between the sixth and eighth months of gestation. In studies conducted in Brazil, abortion rates ranged from 30% to 50% in unvaccinated herds infected with brucellosis (Poester et al., 2013). More recent studies (2020–2023) report abortion rates ranging from 5% to 25% in herds infected with *Brucella abortus*, particularly in regions with limited sanitary control (Blasco et al., 2023), which was later confirmed by Şevik et al., 2025, in molecular tests when they detected *Brucella* spp. in 15.2% (19/125) of aborted bovine fetuses.

Bovine leptospirosis, caused by different serovars of *Leptospira interrogans*, can occur at any stage of gestation; however, abortions are most observed between the fifth and seventh months (Givens and Marley, 2008). Abortion rates can vary significantly depending on the region and the prevalence of specific serovars. According to Ellis (2015), these rates range from 5% to 10% in infected herds, depending on the virulence of the strain and environmental conditions. Later studies reported rates as high as 21.4% in Switzerland (Vidal et al., 2017). In a large study conducted in Canada, where the hardjo-bovis type is prevalent, approximately 6% of abortions were attributed to leptospirosis (Prescott et al., 1988), while a smaller study in the United States identified the disease as the cause of 10% of abortions (Kirkbride and Johnson, 1989). Clinical signs may include infertility, repeat estrus, and sporadic abortions, with abortion rates reaching up to 40% in severely affected herds (Givens and Marley, 2008).

Bovine genital campylobacteriosis (GBC), caused by *Campylobacter fetus* subsp. *fetus* and subsp. *venerealis*, is an infectious disease primarily transmitted through natural mating. It affects the reproductive tract of cows, leading to temporary infertility, early embryonic death, endometritis, and abortion occurring between the fourth and seventh months of gestation. Bulls are asymptomatic and carry the pathogen in their preputial smegma (Campos-Múzquiz et al., 2019; Mughini-Gras et al., 2021). However, fewer than 10% of infected females experience abortion (Garcia and Brooks, 1993), as also supported by recent findings in which Campylobacter spp. accounted for 3.2% (4/125) of bovine abortion cases diagnosed through molecular testing (Şevik, 2025).

Vaccination is a central tool for reducing infectious reproductive losses in beef and dairy cattle. Studies show that the administration of multivalent vaccines against BoHV-1, BVDV, and *Leptospira* spp. prior to TAI reduces pregnancy losses and improves conception rates in beef herds (Aono et al., 2013). A meta-analysis further shows that vaccination against BVDV can reduce abortion by approximately 45% and fetal infection by up to 85% (Newcomer et al., 2015). Despite these benefits, there are no universally standardized reproductive vaccination protocols across countries, production systems, or animal categories, which contributes to the high heterogeneity of results observed under field conditions.

**Figure 1:**
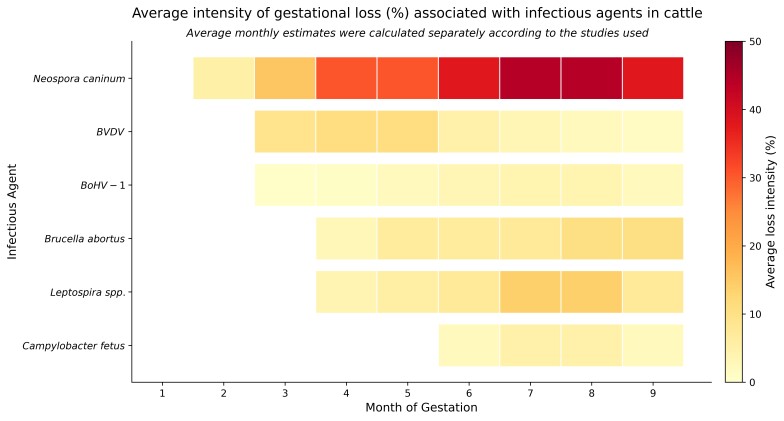
Average of loss intensity (%) caused by infectious agents in cattle.

The Figure 1 illustrates the magnitude of gestational losses caused by the main infectious agents during pregnancy in cattle. Understanding these “risk windows” and the relative impact of each pathogen is essential for informing herd health strategies, such as vaccination protocols and epidemiological monitoring. This knowledge is also valuable for training field personnel, allowing for early detection of clinical signs and rapid outbreak response. Moreover, it supports the implementation of complementary measures, such as biosecurity practices, by focusing efforts on the most critical periods of gestation.

This heatmap summarizes monthly pregnancy loss intensity reported in the literature for major infectious agents. Warmer colors indicate higher estimated loss intensity, based on the gestational periods most affected by each pathogen. Values were extracted from published studies and represent pathogen-specific risk patterns rather than absolute epidemiological estimates. This figure was generated based on the review of articles listed in Table 1 using a Python program via ChatGPT AI (version 5.2).

References used to generate the heatmap: Clothier and Anderson (2016); Cvetojević et al. (2016); Pessoa et al. (2016); Wilson et al. (2016); Derdour et al. (2017); Delooz et al. (2017); Kaveh et al. (2017); Vidal et al. (2017); Díaz-Cao et al. (2018); Moroni et al. (2018); Morrell et al. (2019); Açici et al. (2019); Serrano-Martínez et al. (2019); Lefkaditis et al. (2020); Macías-Rioseco et al. (2020); Zhang et al. (2020); Jonker and Michel (2021); Szeredi et al. (2020); Wolf-Jäckel et al. (2020); Van Loo et al. (2021); Perotta et al. (2021); Villa et al. (2021); Şevik (2021); Ntivuguruzwa et al. (2022); Thomas et al. (2022); Selim et al. (2023); Kim et al. (2024).

It therefore serves as a practical tool for bridging scientific knowledge and field application, assisting farmers, veterinarians, and herd managers in understanding the dynamics of reproductive losses. This, in turn, contributes to improved animal science performance and enhances the sustainability of production systems.

## Final considerations

Gestational losses in cattle range from early and late embryonic mortality to fetal mortality. Most of the records of gestational losses caused by reproductive diseases are in the second and third thirds of pregnancy, which relates to the difficulty in making early diagnoses of losses, especially in the first month of pregnancy, and the biology of the agents involved. Among the infectious agents evaluated, *N. caninum* stood out as the main cause of losses, with high abortion rates recorded in several countries. Other agents such as BVDV, *Leptospira* spp., BoHV-1 and *Campylobacter* fetus also had a significant impact, with variation in loss rates depending on the region and type of production system.

When relating losses to gestational period, we observed greater intensity in the middle and final thirds of gestation. These findings highlight the importance of continuous reproductive monitoring, such as gestational diagnosis at the end of the reproductive season, and the improvement of biosecurity programs.

## Data Availability

Research data is available in the body of the article.

## References

[B001] Açici M, Bölükbaş CS, Pekmezci̇ GZ, Gürler H, Genç O, Gürler AT, Kaya S, Umur Ş (2019). A diagnostic survey of *Neospora caninum* infection in aborted fetuses in the Middle Black Sea Region and Sivas Province, Turkey. Turk J Vet Anim Sci.

[B002] Albaaj A, Durocher J, LeBlanc SJ, Dufour S (2022). Meta-analysis of the incidence of pregnancy losses in dairy cows at different stages to 90 days of gestation. JDS Commun.

[B003] Alves RL, Silva MA, Consentini CE, Silva LO, Folchini NP, Oliva AL, Prata AB, Gonçalves JRS, Wiltbank MC, Sartori R (2021). Hormonal combinations aiming to improve reproductive outcomes of Bos indicus cows submitted to estradiol/progesterone-based timed AI protocols. Theriogenology.

[B004] Andrade JS, Moreira EM, Silva GM, Schneider A, Nunes VRR, Silva RR, Pfeifer LFM (2021). Uterine health and fertility of timed AI postpartum Nelore beef cows raised in the Amazon biome. Livest Sci.

[B005] Andrade MF, Simões J (2024). Embryonic and fetal mortality in dairy cows: incidence, relevance, and diagnosis approach in field conditions. Dairy..

[B006] Antoniassi NA, Juffo GD, Santos AS, Pescador CA, Corbellini LG, Driemeier D (2013). Causas de aborto bovino diagnosticadas no Setor de Patologia Veterinária da UFRGS de 2003 a 2011. Pesq Vet Bras.

[B007] Aono FH, Cooke RF, Alfieri AA, Vasconcelos JL (2013). Effects of vaccination against reproductive diseases on reproductive performance of beef cows submitted to fixed-timed AI in Brazilian cow-calf operations. Theriogenology.

[B008] Ata A, Kocamüftüoğlu M, Hasircioğlu S, Kale M, Gülay MS (2012). Investigation of relationship between Bovine Herpesvirus-1 (BHV-1) infection and fertility in repeat breeding dairy cows in family-type small dairy farms. Kafkas Univ Vet Fak Derg.

[B009] Austin FW, Hopper RM (2021). Bovine reproduction.

[B010] Aymée L, Mendes J, Lilenbaum W (2024). Bovine genital leptospirosis: an update of this important reproductive disease. Animals.

[B011] Baruselli PS, Ferreira RM, Colli MH, Elliff FM, Sá MF, Vieira L, Freitas BG (2017). Timed artificial insemination: current challenges and recent advances in reproductive efficiency in beef and dairy herds in Brazil. Anim Reprod.

[B012] Blasco JM, Moreno E, Muñoz PM, Conde-Álvarez R, Moriyón I (2023). A review of three decades of use of the cattle brucellosis rough vaccine *Brucella abortus* RB51: myths and facts. BMC Vet Res.

[B013] Campos-Múzquiz LG, Méndez-Olvera ET, Arellano-Reynoso B, Martínez-Gómez D (2019). *Campylobacter fetus* is internalized by bovine endometrial epithelial cells. Pol J Microbiol.

[B014] Clothier K, Anderson M (2016). Evaluation of bovine abortion cases and tissue suitability for identification of infectious agents in California diagnostic laboratory cases from 2007 to 2012. Theriogenology.

[B015] Consentini CEC, Alves RLOR, Silva MA, Galindez JPA, Madureira G, Lima LG, Gonçalves JRS, Wiltbank MC, Sartori R (2023). What are the factors associated with pregnancy loss after timed-artificial insemination in *Bos indicus* cattle?. Theriogenology.

[B016] Couto SRB, Guerson YB, Ferreira JE, Silva OR, Silenciato LN, Barbero RP, Mello MRB (2019). Impact of supplementation with long-acting progesterone on gestational loss in Nelore females submitted to TAI. Theriogenology.

[B017] Cvetojević Đ, Savić B, Milićević V, Kureljušić B, Jezdimirović N, Jakić-Dimić D, Pavlović M, Spalević L (2016). Prevalence of Bovine herpesvirus type 4 in aborting dairy cows. Pol J Vet Sci.

[B018] Davenport KM, Ortega MS, Johnson GA, Seo H, Spencer TE (2023). Review: implantation and placentation in ruminants. Animal.

[B019] De Vries A (2006). Economic value of pregnancy in dairy cattle. J Dairy Sci.

[B020] Delooz L, Czaplicki G, Houtain JY, Dal Pozzo F, Saegerman C (2017). Laboratory findings suggesting an association between BoHV-4 and bovine abortions in southern Belgium. Transbound Emerg Dis.

[B021] Derdour SY, Hafsi F, Azzag N, Tennah S, Laamari A, China B, Ghalmi F (2017). Prevalence of the main infectious causes of abortion in dairy cattle in Algeria. J Vet Res.

[B022] Díaz-Cao JM, Prieto Lago A, López Lorenzo G, Díaz Fernández P, López Sández CM, Morrondo Pelayo MP, Fernández-Rodríguez G (2018). Broadening the diagnosis panel of reproductive pathogens associated with abortion in ruminants. Span J Agric Res.

[B023] Diskin MG, Parr MH, Morris DG (2011). Embryo death in cattle: an update. Reprod Fertil Dev.

[B024] Diskin MG, Waters SM, Parr MH, Kenny DA (2016). Pregnancy losses in cattle: potential for improvement. Reprod Fertil Dev.

[B025] Donahoe SL, Lindsay SA, Krockenberger M, Phalen D, Šlapeta J (2015). A review of neosporosis and pathologic findings of *Neospora caninum* infection in wildlife. Int J Parasitol Parasites Wildl.

[B026] Dubey JP, Buxton D, Wouda W (2006). Pathogenesis of bovine neosporosis. J Comp Pathol.

[B027] Eidi S, Seifi HA, Sadr S, Zeinali H (2024). Study on fungal contaminants in aborted calves of cattle herds in Iran. Vet Med Sci.

[B028] Ellis WA (2015). Animal leptospirosis. Curr Top Microbiol Immunol.

[B029] Franco G, Reese S, Poole R, Rhinehart J, Thompson K, Cooke R, Pohler K (2020). Sire contribution to pregnancy loss in different periods of embryonic and fetal development of beef cows. Theriogenology.

[B030] Garcia MM, Brooks BW, Prescott JF (1993). Pathogenesis of bacterial infections in animals..

[B031] Givens MD, Marley MS (2008). Pathogens that cause infertility of bulls or transmission via semen. Theriogenology.

[B032] Graham DA (2013). Bovine herpes virus-1 (BoHV-1) in cattle-a review with emphasis on reproductive impacts and the emergence of infection in Ireland and the United Kingdom. Ir Vet J.

[B033] Grooms DL (2006). Reproductive losses caused by bovine viral diarrhea virus and leptospirosis. Theriogenology.

[B034] Hecker YP, González-Ortega S, Cano S, Ortega-Mora LM, Horcajo P (2023). Bovine infectious abortion: a systematic review and meta-analysis. Front Vet Sci.

[B035] Henker LC, Lorenzett MP, Lopes BC, Dos Santos IR, Bandinelli MB, Bassuino DM, Juffo GD, Antoniassi NAB, Pescador CA, Sonne L, Driemeier D, Pavarini SP (2022). Pathological and etiological characterization of cases of bovine abortion due to sporadic bacterial and mycotic infections. Braz J Microbiol.

[B036] Herlina N, Handharyani E, Setiyono A (2025). Pathogen-driven pregnancy loss in dairy cattle: an overview of diagnostic advances and innovations. Open Vet J.

[B037] Jawor P, Mee JF, Stefaniak T (2021). Role of infection and immunity in bovine perinatal mortality: part 2. Fetomaternal response to infection and novel diagnostic perspectives. Animals.

[B038] Jonker A, Michel AL (2021). Retrospective study of bacterial and fungal causes of abortion in domestic ruminants in northern regions of South Africa (2006-2016). Aust Vet J.

[B039] Kaveh A, Merat E, Samani S, Danandeh S, Soltan Nezhad S (2017). Infectious causes of bovine abortion in Qazvin Province, Iran. Arch Razi Inst.

[B040] Khaneabad A, Taktaz T, Goodarzi S, Momtaz H (2023). BoHV-1 affects abortion and progesterone in dairy cows Bovine alphaherpesvirus 1 (BoHV-1) seropositivity, progesterone levels and embryo loss of 30-day-old pregnant dairy cows in Zagros Industrial Dairy Farm in Shahrekord: examination and analysis. Vet Med Sci.

[B041] Kim J, Kim JW, Lee KK, Lee K, Ku BK, Kim HY (2024). Laboratory investigation of causes of bovine abortion and stillbirth in the Republic of Korea, 2014-2020. J Vet Diagn Invest.

[B042] Kirkbride CA, Johnson MW (1989). Serologic examination of aborted ovine and bovine fetal fluids for the diagnosis of border disease, bluetongue, bovine viral diarrhea, and leptospiral infections. J Vet Diagn Invest.

[B043] Knapek KJ, Georges HM, Van Campen H, Bishop JV, Bielefeldt-Ohmann H, Smirnova NP, Hansen TR (2020). Fetal lymphoid organ immune responses to transient and persistent infection with bovine viral diarrhea virus. Viruses.

[B044] Lefkaditis M, Mpairamoglou R, Sossidou A, Spanoudis K, Tsakiroglou M (2020). *Neospora caninum*, A potential cause of reproductive failure in dairy cows from Northern Greece. Vet Parasitol Reg Stud Reports.

[B045] Macías-Rioseco M, Silveira C, Fraga M, Casaux L, Cabrera A, Francia ME, Robello C, Maya L, Zarantonelli L, Suanes A, Colina R, Buschiazzo A, Giannitti F, Riet-Correa F (2020). Causes of abortion in dairy cows in Uruguay. Pesq Vet Bras.

[B046] Maresca C, Scoccia E, Dettori A, Felici A, Guarcini R, Petrini S, Quaglia A, Filippini G (2018). National surveillance plan for infectious bovine rhinotracheitis (IBR) in autochthonous Italian cattle breeds: results of first year of activity. Vet Microbiol.

[B047] Marugan-Hernandez V (2017). *Neospora caninum* and bovine neosporosis: current vaccine research. J Comp Pathol.

[B048] Mathew DJ, Peterson KD, Senn LK, Oliver MA, Ealy AD (2022). Ruminant conceptus-maternal interactions: interferon-tau and beyond. J Anim Sci.

[B049] Mazuz ML, Fish L, Wolkomirsky R, Leibovich B, Reznikov D, Savitsky I, Golenser J, Shkap V (2015). The effect of a live Neospora caninum tachyzoite vaccine in naturally infected pregnant dairy cows. Prev Vet Med.

[B050] Mee JF (2023). Invited review: bovine abortion-Incidence, risk factors and causes. Reprod Domest Anim.

[B051] Mee JF, Hayes C, Stefaniak T, Jawor P (2023). Review: bovine foetal mortality - risk factors, causes, immune responses and immuno-prophylaxis. Animal.

[B052] Mee JF (2020). Investigation of bovine abortion and stillbirth/perinatal mortality - similar diagnostic challenges, different approaches. Ir Vet J.

[B053] Mercadante VRG, Dias NW, Timlin CL, Pancini S (2020). Economic consequences of pregnancy loss in beef cattle. J Anim Sci.

[B054] Miller JM, Van der Maaten MJ, Whetstone CA (1988). Effects of a bovine herpesvirus-1 isolate on reproductive function in heifers: classification as a type-2 (infectious pustular vulvovaginitis) virus by restriction endonuclease analysis of viral DNA. Am J Vet Res.

[B055] Moroni M, Navarro M, Paredes E, Romero A, Alberdi A, Lischinsky T, Moore DP, Campero CM, Uzal FA (2018). Identification of Neospora caninum in aborted bovine fetuses of southern Chile. Braz J Vet Pathol.

[B056] Morrell EL, Campero CM, Cantón GJ, Odeón AC, Moore DP, Odriozola E, Paolicchi F, Fiorentino MA (2019). Current trends in bovine abortion in Argentina. Pesq Vet Bras.

[B057] Mughini-Gras L, Pijnacker R, Coipan C, Mulder AC, Fernandes Veludo A, de Rijk S, van Hoek AHAM, Buij R, Muskens G, Koene M, Veldman K, Duim B, van der Graaf-van Bloois L, van der Weijden C, Kuiling S, Verbruggen A, van der Giessen J, Opsteegh M, van der Voort M, Castelijn GAA, Schets FM, Blaak H, Wagenaar JA, Zomer AL, Franz E (2021). Sources and transmission routes of campylobacteriosis: A combined analysis of genome and exposure data. J Infect.

[B058] Munhoz AK, Cooke RF, Prado CP, Munhoz SK, de Sousa MCG, da Silva VMP, Pohler KG, Cappellozza BI, Vasconcelos JLM (2025). Characterizing pregnancy losses in lactating Holstein cows receiving a fixed-timed artificial insemination protocol. Anim Reprod Sci.

[B059] Newcomer BW, Walz PH, Givens MD, Wilson AE (2015). Efficacy of bovine viral diarrhea virus vaccination to prevent reproductive disease: a meta-analysis. Theriogenology.

[B060] Ntivuguruzwa JB, Kolo FB, Mwikarago EI, van Heerden H (2022). Characterization of Brucella spp. and other abortigenic pathogens from aborted tissues of cattle and goats in Rwanda. Vet Med Sci.

[B061] Oliveira RV, Cooke RF, de Mello GA, Pereira VM, Vasconcelos JLM, Pohler KG (2022). The effect of subclinical endometritis on reproductive performance in postpartum Bos indicus multiparous beef cows. Anim Reprod Sci.

[B062] Otter A (2020). Cattle abortions update. Vet Rec.

[B063] Perotta JH, Freitas BB, Marcom NN, Pescador CA, Pereira CC, Locatelli-Dittrich R, Brum JS, Barros IR (2021). An abortion storm in dairy cattle associated with neosporosis in southern Brazil. Rev Bras Parasitol Vet.

[B064] Pescador CA, Corbellini LG, Oliveira EC, Raymundo DL, Driemeier D (2007). Histopathological and immunohistochemical aspects of *Neospora caninum* diagnosis in bovine aborted fetuses. Vet Parasitol.

[B065] Pessoa GA, Martini AP, Trentin JM, Dalcin VC, Leonardi CE, Vogel FS, Sá MF, Rubin MI, Silva CA (2016). Impact of spontaneous *Neospora caninum* infection on pregnancy loss and subsequent pregnancy in grazing lactating dairy cows. Theriogenology.

[B066] Poester FP, Samartino LE, Santos RL (2013). Pathogenesis and pathobiology of brucellosis in livestock. Rev Sci Tech.

[B067] Prado CP, Cooke RF, Munhoz AK, Munhoz SK, de Sousa MCG, da Silva VMP, Pohler KG, Vasconcelos JLM (2024). Characterizing pregnancy losses in Bos indicus beef females receiving a fixed-timed artificial insemination protocol. Theriogenology.

[B068] Prescott JF, Miller RB, Nicholson VM, Martin SW, Lesnick T (1988). Seroprevalence and association with abortion of leptospirosis in cattle in Ontario. Can J Vet Res.

[B069] Pugliesi G, Bisinotto DZ, Mello BP, Lahr FC, Ferreira CA, Melo GD, Bastos MR, Madureira EH (2019). A novel strategy for resynchronization of ovulation in Nelore cows using injectable progesterone (P4) and P4 releasing devices to perform two timed inseminations within 22 days. Reprod Domest Anim.

[B070] Pugliesi G, Santos FB, Lopes E, Nogueira É, Maio JR, Binelli M (2016). Improved fertility in suckled beef cows ovulating large follicles or supplemented with long-acting progesterone after timed-AI. Theriogenology.

[B071] Reese ST, Franco GA, Poole RK, Hood R, Fernadez Montero L, Oliveira RV, Cooke RF, Pohler KG (2020). Pregnancy loss in beef cattle: A meta-analysis. Anim Reprod Sci.

[B072] Roth JA, Kaeberle ML, Griffith RW (1981). Effects of bovine viral diarrhea virus infection on bovine polymorphonuclear leukocyte function. Am J Vet Res.

[B073] Santos JE, Thatcher WW, Chebel RC, Cerri RL, Galvão KN (2004). The effect of embryonic death rates in cattle on the efficacy of estrus synchronization programs. Anim Reprod Sci.

[B074] Sarangi LN, Tharani N, Polapally S, Rana SK, Thodangala N, Bahekar VS, Prasad A, Chandrasekhar Reddy RV, Surendra KSNL, Gonuguntla HN, Ponnanna NM, Sharma GK (2021). Infectious bovine abortions: observations from an organized dairy herd. Braz J Microbiol.

[B075] Sartori R, Balistrieri M, Silva LO, Consentini CEC, Melo LF, Pontes GCS, Gaitkoski D (2025). Pregnancy loss in cattle with emphasis on embryo transfer programs. Anim Reprod.

[B076] Schweizer M, Peterhans E (2014). Pestiviruses. Annu Rev Anim Biosci.

[B077] Selim A, Alshammari A, Gattan HS, Marzok M, Salem M, Al-Jabr OA (2023). *Neospora caninum* infection in dairy cattle in Egypt: a serosurvey and associated risk factors. Sci Rep.

[B078] Serrano-Martínez ME, Cisterna CAB, Romero RCE, Huacho MAQ, Bermabé AM, Albornoz LAL (2019). Evaluation of abortions spontaneously induced by *Neospora caninum* and risk factors in dairy cattle from Lima, Peru. Rev Bras Parasitol Vet.

[B079] Şevik M (2021). Genomic characterization of pestiviruses isolated from bovine, ovine and caprine foetuses in Turkey: A potentially new genotype of Pestivirus I species. Transbound Emerg Dis.

[B080] Şevik M (2025). Zoonotic abortifacient agents in bovine abortion: diagnostic assessment of 125 cases (2015-2017). Vet Med Sci.

[B081] Sigdel A, Bisinotto RS, Peñagaricano F (2021). Genes and pathways associated with pregnancy loss in dairy cattle. Sci Rep.

[B082] Smith BD, Poliakiwski B, Polanco O, Singleton S, de Melo GD, Muntari M, Oliveira RV, Pohler KG (2022). Decisive points for pregnancy losses in beef cattle. Reprod Fertil Dev.

[B083] Suarez VH, Martínez GM, Wirsh S (2024). Abortion effects on production, reproductive performance, and health of dairy cattle. Acta Vet Eurasia..

[B084] Szeredi L, Dán Á, Malik P, Jánosi S, Hornyák Á (2020). Low incidence of Schmallenberg virus infection in natural cases of abortion in domestic ruminants in Hungary. Acta Vet Hung.

[B085] Thilsted JP, Dubey JP (1989). Neosporosis-like abortions in a herd of dairy cattle. J Vet Diagn Invest.

[B086] Thomas KM, Kibona T, Claxton JR, de Glanville WA, Lankester F, Amani N, Buza JJ, Carter RW, Chapman GE, Crump JA, Dagleish MP, Halliday JEB, Hamilton CM, Innes EA, Katzer F, Livingstone M, Longbottom D, Millins C, Mmbaga BT, Mosha V, Nyarobi J, Nyasebwa OM, Russell GC, Sanka PN, Semango G, Wheelhouse N, Willett BJ, Cleaveland S, Allan KJ (2022). Prospective cohort study reveals unexpected aetiologies of livestock abortion in northern Tanzania. Sci Rep.

[B087] Van Kuijk SJA, Sonnenberg ASM, Baars JJP, Hendriks WH, Cone JW (2015). Fungal treated lignocellulosic biomass as ruminant feed ingredient: a review. Biotechnol Adv.

[B088] Van Loo H, Pascottini OB, Ribbens S, Hooyberghs J, Pardon B, Opsomer G (2021). Retrospective study of factors associated with bovine infectious abortion and perinatal mortality. Prev Vet Med.

[B089] Vidal S, Kegler K, Greub G, Aeby S, Borel N, Dagleish MP, Posthaus H, Perreten V, Rodriguez-Campos S (2017). Neglected zoonotic agents in cattle abortion: tackling the difficult to grow bacteria. BMC Vet Res.

[B090] Villa L, Maksimov P, Luttermann C, Tuschy M, Gazzonis AL, Zanzani SA, Mortarino M, Conraths FJ, Manfredi MT, Schares G (2021). Spatial distance between sites of sampling associated with genetic variation among *Neospora caninum* in aborted bovine foetuses from northern Italy. Parasit Vectors.

[B091] Walker RL (2007). Current therapy in large animal theriogenology.

[B092] Wallace RM, Pohler KG, Smith MF, Green JA (2015). Placental PAGs: gene origins, expression patterns, and use as markers of pregnancy. Reproduction.

[B093] Wedajo MT, Alemayehu L, Tefera Y, Hagos A, Abadi AR (2021). Seroprevalence of infectious bovine rhinotracheitis and brucellosis and their effect on reproductive performance of dairy cattle. J Vet Med Anim Health.

[B094] Weston JF, Heuer C, Williamson NB (2012). Efficacy of a *Neospora caninum* killed tachyzoite vaccine in preventing abortion and vertical transmission in dairy cattle. Prev Vet Med.

[B095] Wilson DJ, Orsel K, Waddington J, Rajeev M, Sweeny AR, Joseph T, Grigg ME, Raverty SA (2016). *Neospora caninum* is the leading cause of bovine fetal loss in British Columbia, Canada. Vet Parasitol.

[B096] Wiltbank MC, Baez GM, Garcia-Guerra A, Toledo MZ, Monteiro PL, Melo LF, Ochoa JC, Santos JE, Sartori R (2016). Pivotal periods for pregnancy loss during the first trimester of gestation in lactating dairy cows. Theriogenology.

[B097] Wolf-Jäckel GA, Hansen MS, Larsen G, Holm E, Agerholm JS, Jensen TK (2020). Diagnostic studies of abortion in Danish cattle 2015-2017. Acta Vet Scand.

[B098] Zhang H, Deng X, Cui B, Shao Z, Zhao X, Yang Q, Song S, Wang Z, Wang Y, Wang Y, Liu Z, Sheng J, Chen C (2020). Abortion and various associated risk factors in dairy cow and sheep in Ili, China. PLoS One.

